# Differential HLA class I subunit (A, B, C heavy chain and β_2_-microglobulin) expression levels in normal tissues

**DOI:** 10.1007/s00428-022-03459-5

**Published:** 2022-11-27

**Authors:** Filippo Ugolini, Anna Szumera-Ciećkiewicz, Gianna Baroni, Gabriella Nesi, Mario Mandalà, Soldano Ferrone, Daniela Massi

**Affiliations:** 1 Department of Health Sciences, Section of Pathological Anatomy, University of Florence, Viale Pieraccini 6, 50139 Florence, Italy; 2 Department of Pathology, Maria Sklodowska-Curie National Research Institute of Oncology, Warsaw, Poland; 3 Diagnostic Hematology Department, Institute of Hematology and Transfusion Medicine, Warsaw, Poland; 4 Unit of Medical Oncology, University of Perugia, Perugia, Italy; 5 Department of Surgery, Massachusetts General Hospital, Harvard Medical School, Boston, MA USA

**Keywords:** HLA class I, HLA-ABC, β2-microglobulin, TMA, Healthy tissues

## Abstract

Human leukocyte antigen (HLA) class I subunit expression level in primary and metastatic lesions has been characterized in many cancer types utilizing formalin-fixed and paraffin-embedded (FFPE) tissue sections as substrates in immunohistochemical reactions. The evaluation of the results of these studies has been hampered by the scant information about HLA class I subunit expression level in normal tissues. To address this unmet need, we have analyzed the HLA class I subunit expression level in FFPE sections of normal tissues.

Two tissue microarray (TMA) blocks were constructed from archived FFPE tissue samples of a wide number of human normal tissues. The expression level of HLA-A, HLA-B, HLA-C heavy chains and β2-microglobulin (β2-M) was evaluated by IHC staining, with mAb HC-A2, mAb HC-10, and mAb NAMB1, respectively. The staining was scored according to its intensity.

According to their staining patterns with the three mAbs tested, normal tissues can be divided into four groups: (i) tissues displaying moderate/strong staining patterns, (ii) tissues displaying barely detectable staining patterns, (iii) tissues displaying differential staining patterns, and (iv) tissues with no detectable staining. The ubiquitous expression pattern for HLA-A, B, C heavy chain and β2-M was found only at the endothelial level; the stroma was negative except for fibroblasts in all the tissues analyzed. Our data suggest that, contrary to the general postulate, HLA class I subunit expression is not detectable in all nucleated cells. This information provides a useful background to evaluate changes in HLA class I subunit expression associated with the malignant transformation of cells.

## Introduction

The impressive clinical responses observed in cancer patients treated with immune checkpoint inhibitors [1] have restored tumor immunologists’ confidence in the ability of patients’ immune system to recognize and eliminate neoplastic cells [2–5].

As a result, there has been a revival of interest in the role of immune surveillance [6, 7] in the pathogenesis and clinical course of malignant diseases as well as in the expression by malignant cells of molecules which mediate their interactions with the host’s immune system [8, 9]. The driving force for these studies is the expectation that the identification and characterization of defects in the structure and/or function of these molecules will contribute to our understanding of the molecular basis of immune escape mechanisms utilized by malignant cells to avoid immune recognition and destruction as well as to the rational design of strategies to counteract them [10–12].

The immunologically relevant molecules analyzed in malignant cells include the β_2_-microglobulin-HLA class I heavy chain complexes since they mediate interactions between cancer cells and the host’s immune system by presenting tumor antigen-derived peptide to cognate cytotoxic T cells [11, 12].

HLA class I antigen expression levels by malignant cells have been analyzed by IHC staining with mAbs [8]. FFPE tissues represent the substrate of choice in these reactions since they provide the most accurate cellular details and allow the use of collections of archival tissues available in the departments of pathology. The fixation procedure dissociates β2M-HLA class I heavy chain complexes. As a result, the expression of HLA class I subunits, but not that of HLA class I complexes and of HLA class I alleles, can be evaluated. Stained tissue sections are scored by microscopic examination.

The analysis of changes in HLA class I subunit expression levels associated with the malignant transformation of cells has suffered from the limited available information about the HLA class I subunit expression levels in normal tissues [13–15]. To address this unmet need, in this study, we have performed a comprehensive analysis of HLA class I subunit expression levels in normal tissues. We have analyzed separately the expression of HLA-A heavy chains and HLA-B and C heavy chains since their expression is controlled by different regulatory mechanisms and they play distinct functional roles in the interactions of cancer cells with immune cells [16, 17]. To this end, we have used as probes mAbs with selective reactivity with the gene products of HLA class I loci, since mAbs recognizing framework epitopes shared by the gene products of HLA class I loci do not detect their differential expression.

## Materials and methods

### Tissues samples

Archived (years 2012–2020) FFPE samples of 40 human tissues were retrospectively collected from the section of pathological anatomy, Department of Health Sciences of the University of Florence; no autopsy specimens in the tissue sampling were included in this study. Hematoxylin and eosin-stained tissue slides were reviewed by two expert pathologists (AS, DM). The use of FFPE sections of human samples was approved by the local ethics committee (#14865_bio) according to the Helsinki Declaration.

### Tissue microarray construction

Two high-density TMAs were prepared from FFPE samples, using two blocks for each tissue analyzed, as previously described [18]. In brief, cylindrical tissue cores of 1.5 mm diameter were punched out from representative areas of each donor block using specialized TMA equipment and arrayed into two new recipient paraffin blocks at defined array coordinates using an automatic system (TMA Grand Master, 3DHistech). To confirm the evaluation of tissues with no detectable staining or showing conflicting results in TMA, samples of the thyroid, liver, pancreas, adrenal gland, urinary bladder, and placenta were re-tested using up to 5 samples of whole slides for each organ.

### Monoclonal antibodies

The mAb HC-A2, a mouse IgG1 [0.3 µg/1 ml], which recognizes β2-M-free HLA-A (excluding -A24), -B7301, and -G heavy chains, the mAb HC-10 a mouse IgG1 [0.3 µg/1 ml], which recognizes β2-M-free HLA-A3, -A10, -A28, -A29, -A30, -A31, -A32, -A33, and all β2m-free -HLA-B (excluding -B5702, -B5804, and -B73) and -HLA-C heavy chain, and the β2-M-specific mAb NAMB1, a mouse IgG1, [1.2 µg /1 ml], were developed as previously described [19, 20]. mAbs were purified from an ascitic fluid by affinity chromatography on a protein G column (GE Healthcare Life Sciences, Pittsburgh, PA). The purity and activity of mAb preparations were monitored by SDS-PAGE and by binding assays with the cognate antigen, respectively. Commercial abs included: Stat 1 (#14,994, clone D1K9Y, 1:1000, rabbit monoclonal, Cell Signaling, Danvers, MA) that recognizes total STAT1 protein and Phospo-Stat1 (#9167, Tyr701, clone 58D6, rabbit monoclonal, Cell Signaling, Danvers, MA) that detects STAT1 protein phosphorylated tyrosine 701.

### Immunohistochemical staining

Tested antibodies, dilutions, and experimental conditions are summarized in Table 1. Briefly, tissue Sects. (3 µm) were incubated with the HLA class I subunit-specific mAbs on a Ventana Discovery Ultra immunostainer (Ventana Medical Systems, Tucson, AZ). Negative controls (mouse and rabbit monoclonal negative control Ig, Vector Laboratories) were simultaneously performed to exclude the presence of any non-specific staining. The staining procedure included pretreatment with cell conditioner 1 (CC1) followed by incubation with the tested antibodies. For all antibodies, the signal was developed with the UltraMap DAB anti-Mouse Detection Kit (Ventana Medical Systems, Tucson, AZ). Sections were then counterstained with hematoxylin.

**Table 1:** Antibodies used for immunohistochemistry

Antibodies target	Clone	Antigen retrieval	Incubation time	Dilution
HLA A	[HC-A2]	32 min with CC1	12 h	1:3000
HLA B, C	[HC-10]	32 min with CC1	1 h	1:3000
β2-Microglobulin	[NAMB-1]	32 min with CC1	2 h	1:800
STAT 1	[D1K9Y]	64 min with CC1	2 h	1:1000
P-STAT1	[58D6]	64 min with CC1	6 h	1:100

Digital image acquisition and analysis of tissue sections stained with the HLA class I subunit-specific mAbs, with the STAT1-specific rabbit mAb, and with the P-STAT1-specific rabbit mAb were digitally scanned at X400 magnification by using the Aperio AT2 platform (Leica Biosystems, Wetzlar, Germany). For each antibody, staining was scored as 0 (not detectable), 1 + (weak intensity), 2 + (moderate intensity), and 3 + (strong intensity). All TMA sections and whole tissue sections were scored by two pathologists. On the basis of clinical records, we can rule out that immune modulation has influenced the HLA class I subunit expression, and the discrepancies in evaluations were resolved by joint discussion with the support of digital image acquisition.

## Results

**Fig. 1:**
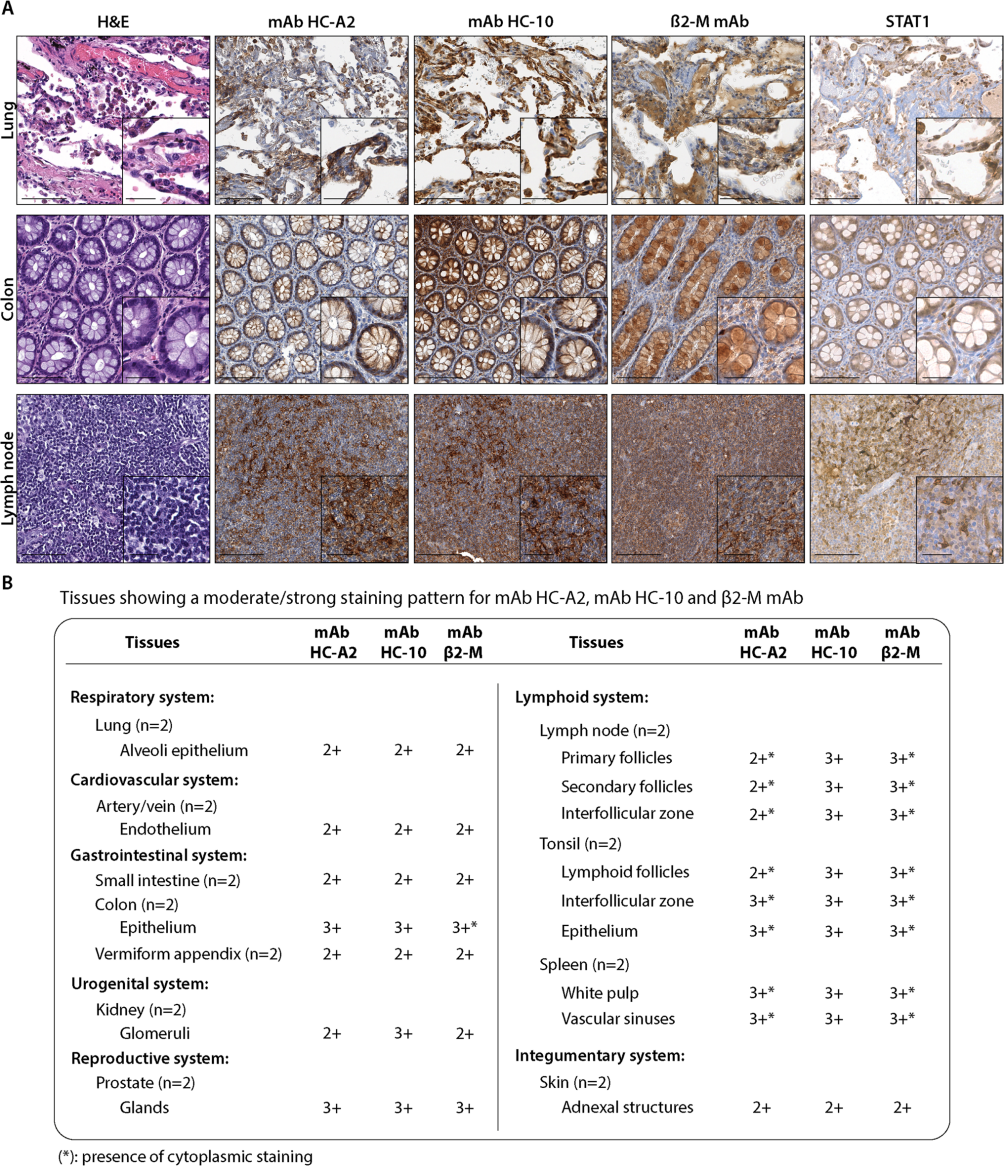
**A** Representative tissue samples showing a moderate/strong staining pattern for mAb HC-A2, mAb HC-10, and β2-M. Immunohistochemical images of lung, colon, and lymph node healthy tissues with mAb HC-A2, mAb HC-10, β2-M, and STAT1. Magnification 200X, inset 400X (scale bars 200 µm, 50 µm, respectively). **B** Summary table of tissues showing a moderate/strong staining pattern for mAb HC-A2, mAb HC-10, and β2 mAb

**Fig. 2:**
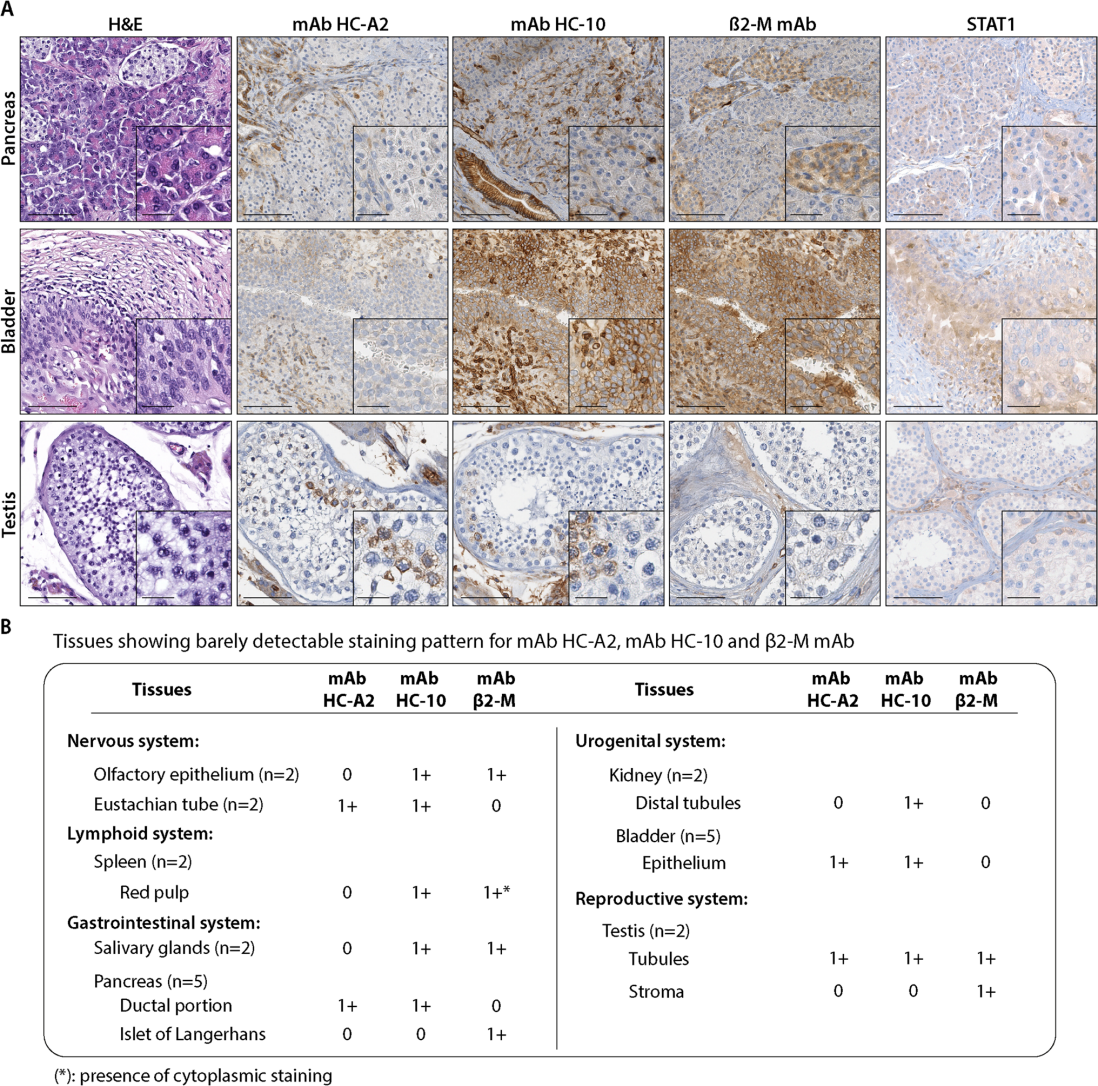
**A** Representative tissue samples showing a barely staining pattern for mAb HC-A2, mAb HC-10, and β2-M. Immunohistochemical images of pancreas, bladder, and testis healthy tissues with mAb HC-A2, mAb HC-10, β2-M, and STAT1. Magnification 200X, inset 400X (scale bars 200 µm, 50 µm, respectively). **B** Summary table of tissues showing a barely detectable staining pattern for mAb HC-A2, mAb HC-10, and β2 mAb

**Fig. 3:**
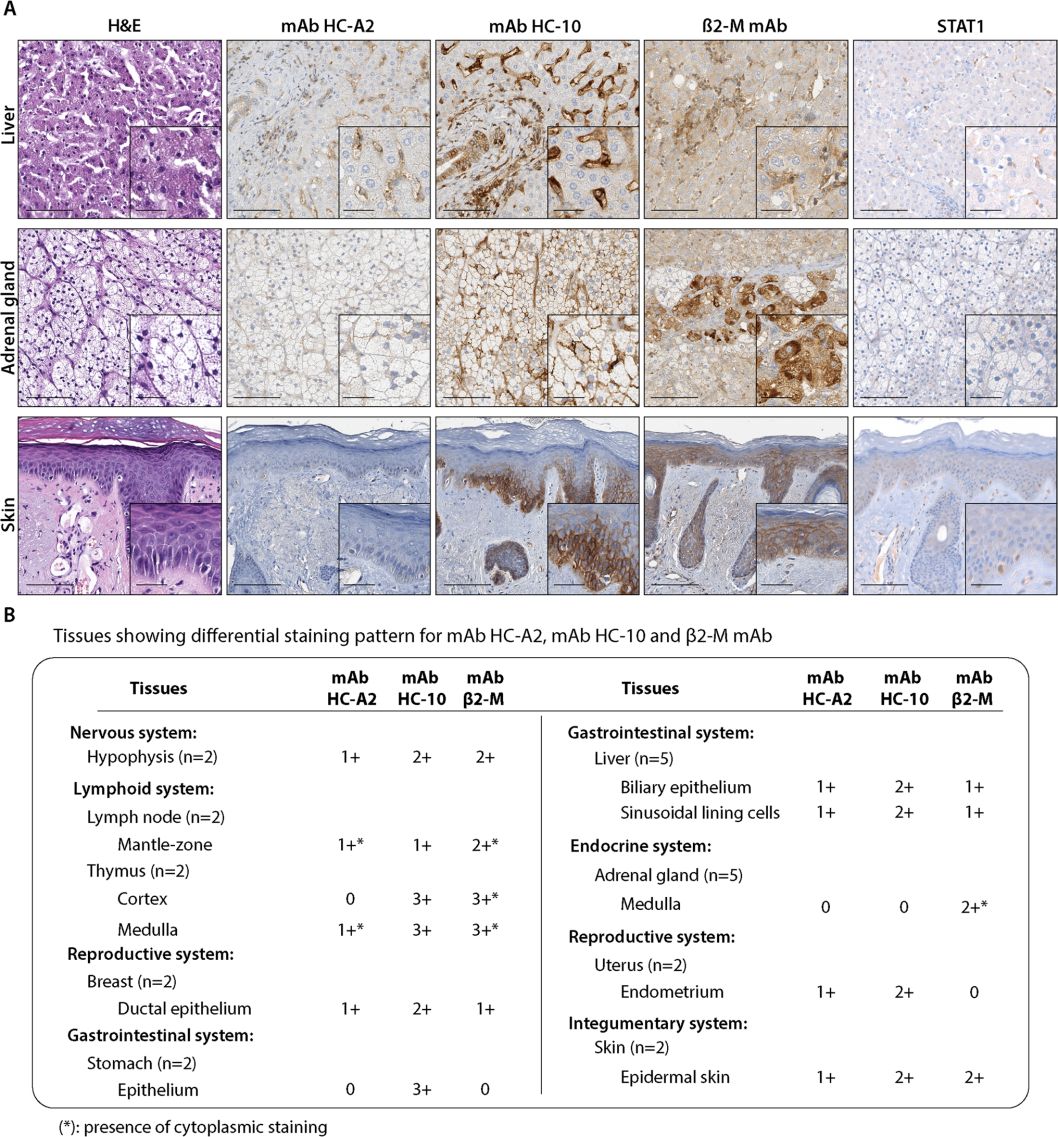
**A** Representative tissue samples showing differential staining pattern for mAb HC-A2, mAb HC-10, and β2-M. Immunohistochemical images of the liver, adrenal gland, and skin-healthy tissues with mAb HC-A2, mAb HC-10, β2-M, and STAT1. Magnification 200X, inset 400X (scale bars 200 µm, 50 µm, respectively). **B** Summary table of tissues showing a differential staining pattern for mAb HC-A2, mAb HC-10, and β2 mAb

**Fig. 4:**
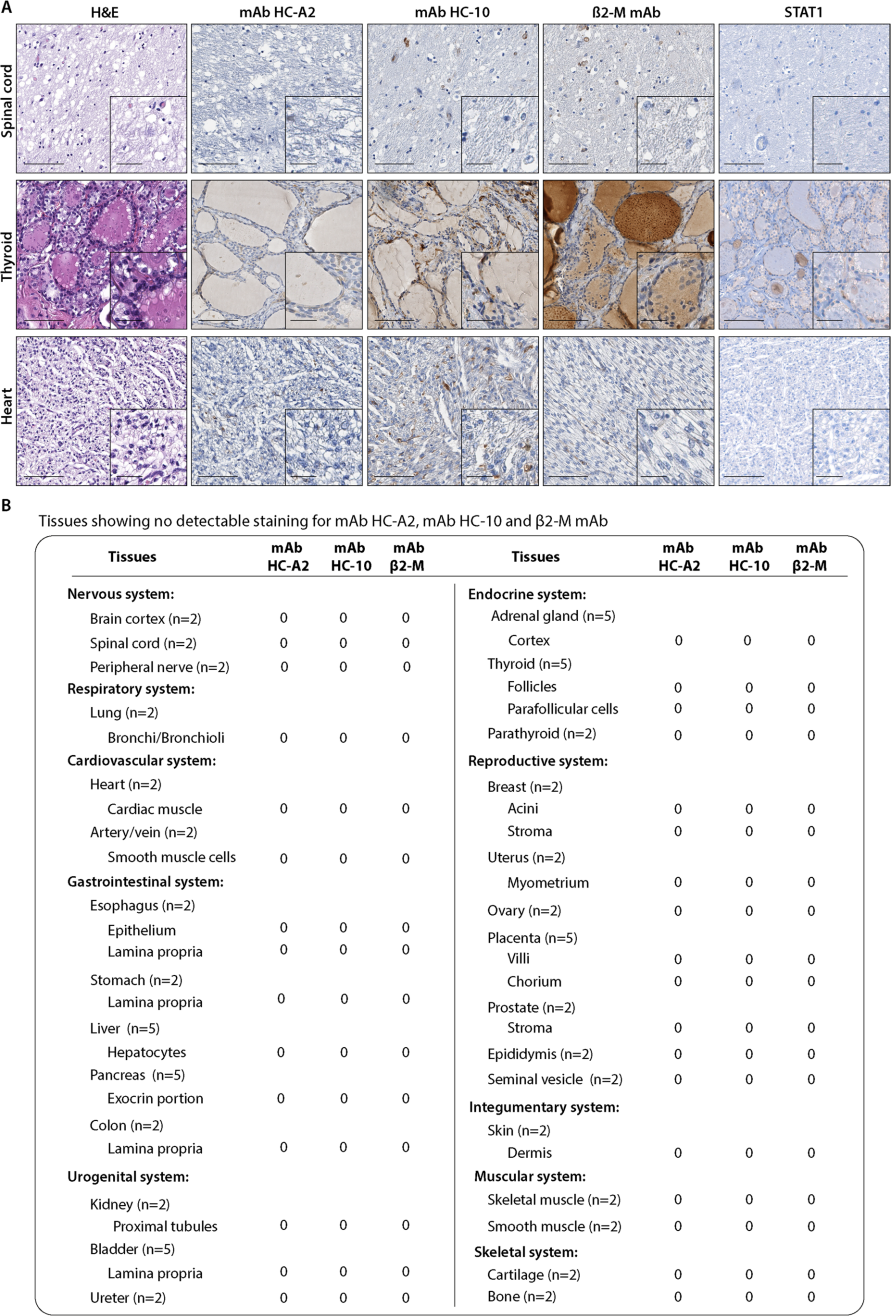
**A** Representative tissue samples showing no detectable staining pattern for mAb HC-A2, mAb HC-10, and β2-M. Immunohistochemical images of the spinal cord, thyroid, and heart-healthy tissues with mAb HC-A2, mAb HC-10, β2-M, and STAT1. Magnification 200X, inset 400X (scale bars 200 µm, 50 µm, respectively). **B** Summary table of tissues showing no detectable staining pattern for mAb HC-A2, mAb HC-10, and β2 mAb

According to their staining patterns with the HLA-A heavy chain-specific mAb HC-A2, the HLA-B, C heavy chain-specific mAb HC-10, and the β2-M mAb NAMB1, tissues can be divided into four groups: (i) tissues displaying moderate/strong staining patterns with the three HLA class I subunit-specific mAbs, (ii) tissues displaying barely detectable staining patterns with the three mAbs, (iii) tissues displaying differential staining patterns with the three mAbs, and (iv) tissues with no detectable staining by the three mAbs (Figs. 1A–4A). We found a ubiquitous expression pattern for HLA-A, B, C heavy chain and β2-M only at the endothelial level that was always positive (moderate staining) across every tissue tested; the stroma was negative except for fibroblasts which were moderately positive in all the tissues analyzed. The staining was restricted to the cell membrane of the nucleated cells in all the tissues tested, except for focal cytoplasmic staining by the HLA-A heavy chain-specific mAb HC-A2 and the β_2_-M-specific mAb NAMB1 in tonsil, thymus, lymph node, spleen, colon epithelium, and adrenal gland medulla.

### Tissues with moderate/strong staining patterns by HLA class I subunit-specific mAbs

This group includes the tonsil, lymph node (except for mantle zone), and spleen (except for red pulp) in the lymphoid system; pulmonary alveolar epithelium in the respiratory system; artery/vein endothelium in the cardiovascular system; the epithelial lining cells of the small intestine, colon, and vermiform appendix in the gastrointestinal system; the glomeruli of the kidney in the urogenital system; prostate glands in the reproductive system; and adnexal structures of skin in the integumentary system (Fig. 1B). Representative examples of the staining patterns are shown in Fig. 1. In this group of tissues, HLA class I subunit expression was associated with the expression of total STAT1 (Fig. 1A). P-STAT1 expression was not detectable (data not shown).

### Tissues with barely detectable staining patterns by HLA class I subunit-specific mAbs

This group includes the olfactory epithelium and Eustachian tube in the nervous system; the red pulp of the spleen in the lymphoid system; the salivary glands, ductal portion, and Langerhans’s islet of the pancreas in the gastrointestinal system; the distal tubules of the kidney in the urogenital system; and the testis in the reproductive system (Fig. 2B). Representative examples of the staining patterns are shown in Fig. 2. Also, in this group, HLA class I subunit expression was associated with the expression total of STAT1 (Fig. 2A). P-STAT1 expression was not detectable (data not shown).

### Tissues with differential staining patterns by HLA class I subunit-specific mAbs

This group includes hypophysis in the nervous system, lymph node’s mantle zone and thymus of in the lymphoid system, breast ductal epithelium in the reproductive system, stomach’s epithelium and liver’s epithelium and sinusoidal lining cells in the gastrointestinal system, medulla of the adrenal gland in the endocrine system, uterus endometrium in the reproductive system, and the epidermal skin in the integumentary system (Fig. 3B). Representative examples of the staining patterns are shown in Fig. 3A. Low/moderate STAT1 staining was detectable in the hypophysis, lymph node’s mantle zone, and uterus. The other tissues of this group, including the thymus, breast, stomach’s epithelium, liver, medulla of the adrenal gland, and skin, were nearly negative with the presence of few positive cells. P-STAT1 expression was not detectable (data not shown).

### Tissues with no detectable staining by HLA class I subunit-specific mAbs

This group includes the brain cortex, spinal cord, and peripheral nerve in the nervous system; bronchi/bronchioli in the respiratory system; heart, artery, and vein in the cardiac system; epithelium, lamina propria of the esophagus, and lamina propria of the colon in the gastrointestinal system; proximal tubules of the kidney, lamina propria of the urinary bladder, and ureter in the urogenital system; thyroid, parathyroid, and cortex of adrenal gland in the endocrine system; acini and stroma of breast, myometrium of uterus, ovary, placenta, the stroma of prostate, epididymis, and seminal vesicles in the reproductive system; dermis of skin in the integumentary system; smooth and skeletal muscle fibers; bone and cartilage in the skeletal system (Fig. 4B). Representative examples of the staining patterns are shown in Fig. 4. Total STAT1 (Fig. 4A) and P-STAT1 (data not shown) expressions were not detectable. In order to exclude that the lack of stain in this large group of tissues was due to antigenic conservation, we compared the staining of FFPE samples 20 years old with FFPE from 2020. We confirmed a high conservation of these antigens demonstrating no differences in staining patterns between samples 20 years old and the more recent ones.

## Discussion

A comprehensive and extensive IHC analysis with mAbs of a large number of FFPE normal tissues has shown that, contrary to what is generally assumed, HLA class I subunits are not expressed by nucleated cells in all the normal tissues [21–23]. Many of them are barely or not stained by mAbs reacting with HLA-A heavy chain, HLA-B heavy chain, HLA-C heavy chain, and β2-M.

Thanks to the availability of innovative and specific antibodies, we propose that healthy human tissues be classified into four different groups, based on the mAb HC-A2, mAb HC-10, and β_2_-M mAb staining intensity and cellular localization: (i) tissues with moderate/strong staining patterns by HLA class I subunit-specific mAbs, (ii) tissues showing a barely detectable staining pattern by HLA class I subunit-specific mAbs, (iii) tissues showing a differential staining pattern by HLA class I subunit-specific mAbs, (iv) and tissues with no detectable staining by HLA class I subunit-specific mAbs.

Our results parallel those obtained by testing normal frozen tissues [13–15] with mAb PA 2.6 and mAb 06/64 for HLA ABC highlighting some differences. Differences include the use of frozen *vs.* FFPE tissues [13–15], antibodies with diverse sensitivity, and methodological variability in the employed assays. In particular, previous studies used a manual peroxidase-antiperoxidase (PAP) method with the monoclonal antibody PA2.6, which reacts similarly to w6/32 that recognizes an epitope on the HLA-A,B,C heavy chain/β2 micro-globulin complex in the membrane of cells [13–16]. While in the present study, we employed a more accurate and sensitive automated IHC procedure, and we used monoclonal antibodies with higher specificity that although they are unable to detect the HLA-A,B,C heavy chain/β2 micro-globulin complex, they allowed us to discriminate the subcellular localization of the single sub-units in FFPE tissues [24, 25].

Another point of strength of our study was the use of TMA, which is a rapid and high-throughput technique to assay numerous tissues arrayed on a single slide [26]. TMA allows a reliable semiquantitative scoring of the intensity of the staining because all tissue samples on a TMA slide are exposed to the same amount of primary and secondary antibody and chromogen. Simultaneous analysis of a large number of specimens decreases time and cost since only a small amount of each reagent is needed to assay all cores at the same time. A potential caveat is a more comprehensive analysis of tissues in the presence of tissue heterogeneity, particularly for small cores. Nevertheless, by using normal and not tumoral tissues, this could represent a partial boundary; however, we re-tested on the whole section of every sample that was negative for the three staining or not evaluable on TMAs.

The reported data clearly showed that the tissue distribution of HLA A, B, C heavy chains and β_2_-M is not uniform all over the tested tissues. In this context, our study is relevant and timely by providing the most extensive and comprehensive evaluation of HLA class I subunit expression (A, B, C heavy chains and β_2_-M) in different human healthy tissues. This represents a backbone analysis for future functional studies in cancer tissues with the aim to elucidate the baseline staining expression of HLA A, B, C heavy chains and β_2_-M in healthy tissues. The expression of the gene products of HLA class I loci is controlled by different regulatory mechanisms [16]. One of the most important pathways is interferon-γ (IFN-γ)/Janus kinase (JAK)/signal transducer and activator of transcription (STAT1). The IFN-γ/JAK/STAT1 pathway plays a crucial role in the antigen processing pathway and the subsequent dynamic change of downstream signals, including the HLA class I subunit [16, 27]. The differential basal expression of HLA A, B, C heavy chain and β_2_-microglobulin in normal tissues could be justified by the parallel differential basal expression of the transcription factor STAT1. In this paper, we show that there is an interesting association between the expression of HLA A, B, C heavy chains and β_2_-M and the expression of STAT1.

Although these results strongly support our thesis that HLA class I subunits are not ubiquitously expressed, on the other hand, we expected to find differences in the levels of phosphorylated STAT1 as well. Instead, all healthy tissues tested were negative. This unexpected result may be due to the basal activity of STAT1. Activated in response to many different cytokines and growth factors by phosphorylation of specific tyrosine residues, STAT1 enhances its transcription factor effect. This strong activation has been shown to be present in many pathological conditions such as injury, ischemia, and tumors [28–30]. We could assume that in healthy tissues, this activation does not occur and that the non-phosphorylated form STAT1 handles the basal expression of proteins such as HLA class I components [31].

Expression of HLA class I subunits in healthy human tissues should be considered when evaluating the increase, reduction, or loss of HLA I expression on malignant cells reported in many types of cancer [32–35]. The amount of these molecules expressed at the cell surface varies significantly depending on the level of gene transcription, transduction, and epigenetic regulation (8). In immunohistochemical studies, however, the boundary between complete, irreversible loss, and down-regulation (or low expression) of HLA in tumor tissues may be unclear if the baseline level of expression in the respective normal tissue is not taken into consideration.

In conclusion, our data elucidated the selective distribution and patterns of expression of HLA A, B, C heavy chains and β_2_-microglobulin in healthy human tissues. We showed that the expression of the three main subunits of the HLA class I is tissue-specific and can vary within the same districts, emphasizing again the caution that is needed to explain the changing levels of expression of these antigens in respective tumor tissues. Thus, our data could add new insight for the interpretation of immunohistochemical studies in different cancer types, and it may improve the understanding of the mechanisms of escape adopted by malignant cells during tumor development and progression.

## Data Availability

All the data analyzed during this study is included in this article.

## References

[1] Sharma P, Allison JP (2015). The future of immune checkpoint therapy. Science.

[2] Srivastava RM, Lee SC, Andrade Filho PA (2013). Cetuximab-activated natural killer and dendritic cells collaborate to trigger tumor antigen-specific T-cell immunity in head and neck cancer patients. Clin Cancer Res.

[3] Sette A, Chesnut R, Fikes J (2001). HLA expression in cancer: implications for T cell-based immunotherapy. Immunogenetics.

[4] Leko V, Rosenberg SA (2020). Identifying and targeting human tumor antigens for T cell-based immunotherapy of solid tumors. Cancer Cell.

[5] Chang CC, Ferrone S (2007). Immune selective pressure and HLA class I antigen defects in malignant lesions. Cancer Immunol Immunother.

[6] Campoli M, Ferrone S (2011). HLA antigen and NK cell activating ligand expression in malignant cells: a story of loss or acquisition. Semin Immunopathol.

[7] Campoli M, Ferrone S (2008). HLA antigen changes in malignant cells: epigenetic mechanisms and biologic significance. Oncogene.

[8] Seliger B (2014). The link between MHC class I abnormalities of tumors, oncogenes, tumor suppressor genes, and transcription factors. J Immunotoxicol.

[9] Campoli M, Ferrone S, Zea AH (2005). Mechanisms of tumor evasion. Cancer Treat Res.

[10] Ferris RL, Whiteside TL, Ferrone S (2006). Immune escape associated with functional defects in antigen-processing machinery in head and neck cancer. Clin Cancer Res.

[11] Ferrone S, Whiteside TL (2007). Tumor microenvironment and immune escape. Surg Oncol Clin N Am.

[12] Bangia N, Ferrone S (2006). Antigen presentation machinery (APM) modulation and soluble HLA molecules in the tumor microenvironment: Do they provide tumor cells with escape mechanisms from recognition by cytotoxic T lymphocytes?. Immunol Invest.

[13] Fleming KA, McMichael A, Morton JA (1981). Distribution of HLA class 1 antigens in normal human tissue and in mammary cancer. J Clin Pathol.

[14] Daar AS, Fuggle SV, Fabre JW (1984). The detailed distribution of HLA–A, B, C antigens in normal human organs. Transplantation.

[15] Natali PG, Nicotra MR, Viora M et al (1984) Distribution of human class I (HLA-A, B, C) histocompatibility antigens in normal and malignant tissues of nonlymphoid origin. In: Cancer Res. https://pubmed.ncbi.nlm.nih.gov/6590117/6590117

[16] Jongsma MLM, Guarda G, Spaapen RM (2019). The regulatory network behind MHC class I expression. Mol Immunol.

[17] Pellegrino MA, Ng AK, Russo C, Ferrone S (1982). Heterogeneous distribution of the determinants defined by monoclonal antibodies on HLA-A and B antigens bearing molecules. Transplantation.

[18] Zlobec I, Suter G, Perren A, Lugli A (2014). A next-generation tissue microarray (ngTMA) protocol for biomarker studies. J Vis Exp.

[19] Stam NJ, Vroom TM, Peters PJ (1990). HLA-A- and HLA-B-specific monoclonal antibodies reactive with free heavy chains in western blots, in formalin-fixed, paraffin-embedded tissue sections and in cryo-immuno-electron microscopy. Int Immunol.

[20] Perosa F, Luccarelli G, Prete M (2003). β 2 -microglobulin-free HLA class I heavy chain epitope mimicry by monoclonal antibody HC-10-specific peptide. J Immunol.

[21] Berah M, Hoes J, Dausset J (1970). A study of hl-a antigens in human organs. Transplantation.

[22] Williams KA, Hart DNJ, Fabre JW, Morris PJ (1980). Distribution and quantitation of hla-abc and dr (ia) antigens on human kidney and other tissues. Transplantation.

[23] Bodmer WF (1981). HLA structure and function: a contemporary view. Tissue Antigens.

[24] Biesterfeld S, Kraus HL, Reineke T, et al (2003) Analysis of the reliability of manual and automated immunohistochemical staining procedures: a pilot study. In: Anal. Quant. Cytol. Histol. https://pubmed.ncbi.nlm.nih.gov/12746978/12746978

[25] Trautz F, Dreßler J, Stassart R (2018). Proposals for best-quality immunohistochemical staining of paraffin-embedded brain tissue slides in forensics. Int J Legal Med.

[26] Skacel M, Skilton B, Pettay JD, Tubbs RR (2002). Tissue microarrays: a powerful tool for high-throughput analysis of clinical specimens. Appl Immunohistochem Mol Morphol.

[27] Groothuis TAM, Griekspoor AC, Neijssen JJ (2005). MHC class I alleles and their exploration of the antigen-processing machinery. Immunol Rev.

[28] Zhang Y, Liu Z (2017) STAT1 in cancer: friend or foe? In: Discov Med. https://pubmed.ncbi.nlm.nih.gov/28950072 28950072

[29] Meissl K, Macho-Maschler S, Müller M, Strobl B (2017). The good and the bad faces of STAT1 in solid tumours. Cytokine.

[30] Butturini E, Boriero D, Carcereri de Prati A, Mariotto S (2019). STAT1 drives M1 microglia activation and neuroinflammation under hypoxia. Arch Biochem Biophys.

[31] Yang J, Stark GR (2008). Roles of unphosphorylated STATs in signaling. Cell Res.

[32] McDougall CJ, Ngoi SS, Godwin T, et al (1990) Reduced expression of HLA class I and II antigens in colon cancer. In: Cancer Res. https://pubmed.ncbi.nlm.nih.gov/2123744/2123744

[33] Garcia-Lora A, Martinez M, Algarra I (2003). MHC class I-deficient metastatic tumor variants immunoselected by T lymphocytes originate from the coordinated downregulation of APM components. Int J Cancer.

[34] Giorda E, Sibilio L, Martayan A, et al (2003) The antigen processing machinery of class I human leukocyte antigens: linked patterns of gene expression in neoplastic cells. In: Cancer Res. https://pubmed.ncbi.nlm.nih.gov/12874016 12874016

[35] Ichinokawa K, Nakanishi Y, Hida Y (2019). Downregulated expression of human leukocyte antigen class I heavy chain is associated with poor prognosis in non-small-cell lung cancer. Oncol Lett.

