# Correction to: Differential HLA class I subunit (A, B, C heavy chain and β2-microglobulin) expression levels in normal tissues

**DOI:** 10.1007/s00428-024-03750-7

**Published:** 2024-02-05

**Authors:** Filippo Ugolini, Anna Szumera-Ciećkiewicz, Gianna Baroni, Gabriella Nesi, Mario Mandalà, Soldano Ferrone, Daniela Massi

**Affiliations:** 1 Department of Health Sciences, Section of Pathological Anatomy, University of Florence, Viale Pieraccini 6, 50139 Florence, Italy; 2 Department of Pathology, Maria Sklodowska-Curie National Research Institute of Oncology, Warsaw, Poland; 3 Diagnostic Hematology Department, Institute of Hematology and Transfusion Medicine, Warsaw, Poland; 4 Unit of Medical Oncology, University of Perugia, Perugia, Italy; 5 Department of Surgery, Massachusetts General Hospital, Harvard Medical School, Boston, MA USA

**Correction to: Virchows Archiv (2022) 482:359-368**

10.1007/s00428-022-03459-5

In this article, the reference citations were incorrectly arranged and has been replaced with the correct citations.

The original article has been corrected.

